# Generosity motivated by acceptance - evolutionary analysis of an anticipation game

**DOI:** 10.1038/srep18076

**Published:** 2015-12-14

**Authors:** I. Zisis, S. Di Guida, T. A. Han, G. Kirchsteiger, T. Lenaerts

**Affiliations:** 1 MLG, Département d’Informatique, Université Libre de Bruxelles, Brussels, Belgium; 2 Department of Business and Economics, COHERE, Syddansk Universitet, Odense, Denmark; 3 School of Computing, Teesside University, Middlesbrough, UK; 4 ECARES, SBS-EM, Université Libre de Bruxelles, Brussels, Belgium; 5 AI lab, Computer Science Department, Vrije Universiteit Brussel, Brussels, Belgium

## Abstract

We here present both experimental and theoretical results for an Anticipation Game, a two-stage game wherein the standard Dictator Game is played after a matching phase wherein receivers use the past actions of dictators to decide whether to interact with them. The experimental results for three different treatments show that partner choice induces dictators to adjust their donations towards the expectations of the receivers, giving significantly more than expected in the standard Dictator Game. Adding noise to the dictators’ reputation lowers the donations, underlining that their actions are determined by the knowledge provided to receivers. Secondly, we show that the recently proposed stochastic evolutionary model where payoff only weakly drives evolution and individuals can make mistakes requires some adaptations to explain the experimental results. We observe that the model fails in reproducing the heterogeneous strategy distributions. We show here that by explicitly modelling the dictators’ probability of acceptance by receivers and introducing a parameter that reflects the dictators’ capacity to anticipate future gains produces a closer fit to the aforementioned strategy distributions. This new parameter has the important advantage that it explains where the dictators’ generosity comes from, revealing that anticipating future acceptance is the key to success.

Literature on biological markets^1^,^2^,^3^ and competitive altruism^4^,^5^ has revealed that partner choice (or selection) provides an important mechanism to explain the sustained levels of cooperation and fairness within the context of social dilemmas. Partner selection is described as a method that allows individuals belonging to one group to select a partner among the members of another group, preferentially attaching to those that are considered to be the most advantageous for the situation. As the potential partners are fully aware of this selection process they will, when required, adapt their behaviour in order to become more attractive, generating in a sense competition among themselves.

A number of biological market models were recently introduced to explain the generosity typically observed in the context of gift-giving games like the Dictator Game (DG) and modified versions of it^6^. In the DG^7^,^8^, a dictator receives an endowment that she has to divide between herself and the other player, the receiver. In the standard format of the game the receiver cannot take any action, obtaining whatever the dictator wishes to give. Literature shows that in that scenario more than 60% of subjects pass a positive amount of money, with a mean transfer of approximately 28% of the endowment^9^,^10^. Whether this non-zero amount is given for altruistic or selfish reasons is still under debate^11^. Nonetheless, the DG shows that human populations are more generous than what is expected from a population of rational and selfish payoff maximising individuals. The aforementioned biological market models argue that this dictator generosity is the result of a combination of partner selection, the demography of the two classes of individuals (dictators and receivers) and the resource division. They furthermore contrasted their work with the analysis performed by Nowak and colleagues^12^, arguing that the balanced outcome observed in that case, which is defined in the context of the Ultimatum Game (UG), is a consequence of an a priori restriction of the parameter space and would have lead to an unfair outcome in favour of the receivers without that restriction. They question the evidence provided by this model for the importance of reputation as a proximate explanation for the evolution of generous behaviour.

Here we go beyond this prior work by first providing experimental insights into how humans behave in a modified DG wherein prior to playing that game receivers have the possibility to decide whether they accept a given dictator, using information on the dictator’s actions in previous rounds to make that decision. Afterwards we provide an evolutionary explanation of the observed behaviour through the highly parsimonious stochastic evolutionary model proposed by Rand *et al.*^13^, showing that in order to identify the origins of the generosity observed in the different experiments one needs to consider that dictators have acquired the capacity to anticipate. Our extension to the Rand *et al.* stochastic model will ensure a better match with the individual-level behaviour, which is, as was also argued by Fowler and Christakis^14^, a weakness of the stochastic model.

Thus prior to playing the DG, a receiver can decide whether or not to play the game with a proposed dictator. Receivers do not select their preferred partner among the full set of dictators as is assumed in biological markets. Neither do we use specific matching algorithms^15^. At each round receivers and dictators are paired-up randomly and then each receiver decides whether she wishes to play the DG with the suggested dictator or not. By accepting the proposed dictator, both players will gain a payoff equal to the amount that they will receive from playing the game, whereas, when choosing to reject the proposed dictator both players will gain zero payoff. On one hand, this game follows a basic model discussed by André and Baumard^6^, yet simplifies the receivers’ choice to either accepting or rejecting a proposed dictator. This form of partner selection thus reduces the mechanism to its weakest form, providing a lower limit of what can be expected in terms of donation levels within this context. On the other hand, our experiments highlight the role of reputation for the generosity of dictators since receivers will decide to accept dictators based on a variety of social cues. This setup differs from the reputation-based model discussed by Nowak and colleagues^12^ where the reputation was assigned to receivers. As the dictator is aware that a future matched receiver will obtain certain social clues about her past, she may anticipate how to behave in order to be a more attractive partner, while at the same time benefiting the most from this social interaction. No experimental results exist that show the level of dictator generosity and the probability of receiver acceptance in this kind of game, which we will refer to as the Anticipation Game (AG) in the remainder of this text.

We aim to show experimentally how different social cues influence the level of dictator donations in the AG as well as the heterogeneous distribution of dictator strategies one can observe. Especially the latter is of interest as it provides insights into a diversity of human generosity levels^10^,^16^, which range from players that keep the maximum of the endowment even when this affects their social reputation to individuals that give more with varying degrees of generosity. Prior modelling work pays little attention to this heterogeneity. It was only recently suggested that the variation and imbalance in donations may be due to how dictators perceive the ownership of the initial endowment^3^.

Four different treatments are performed here: A baseline treatment (treatment 1) that re-examines the DG, which is used for comparison, and three AG treatments wherein receivers approve their matching with a certain dictator based on different pieces of information. Every AG treatment consists of 30 rounds, wherein dictators and receivers are matched randomly. In every round, the receiver will need to decide whether she wishes to accept or not the given dictator using information about the past actions or reputation of her proposed dictator. When the receiver refuses to play both individuals will, as mentioned earlier, receive zero payoff and have to wait for the next round of the game. The dictator, knowing that what she gives now might be observable by receivers in the future, has to decide how much to give from the endowment of 10 Experimental Currency Units (ECUs) she is given at the start of every round, with the smallest donation equal to 1. The minimum of 1 ECU is required as a donation of 0 ECU would create 2 subgame perfect equilibria (see Figure S1 in Supplementary Material) and hence a receiver could be indifferent between playing or not playing the game. As the dictator knows that the experiment takes multiple rounds, where in each round her past actions will be made explicit to the receiver, this voluntary matching introduces an explicit strategic concern about future interactions for the dictator.

The three AG treatments differ in the information provided to the receiver after the initiation phase (see Methods and Supplementary Material). In the first AG treatment (treatment 2), the receivers can observe only the amounts given in the last three rounds when the dictator was accepted to play and use it to decide whether to play or not with her. In the second AG treatment (treatment 3), information on the number of times a dictator was refused by receivers was also added to the history, providing additional information on his or her reputation. In the last AG treatment (treatment 4 or noisy AG), which is also an adaptation of treatment 2, there is 50% chance in each round that a receiver does not know the amounts given by the matched dictator, having to make an uninformed decision on whether to play or not play with the given dictator. Dictators were informed about the presence or absence of this information for the receiver. Next to the average donation levels and strategy distributions we analyse also the receivers’ acceptance rate in order to understand when a dictator is likely to be considered as partner. The results of the noisy AG experiment will reveal the importance of having information on the dictator’s reputation for the donation levels.

As argued earlier, we provide also an evolutionary explanation for the origins of the observed results different from the biological market mechanisms provided in the literature. We adopt a stochastic evolutionary model^13^, as opposed to a deterministic infinite population dynamics model^6^. The idea of this stochastic model is to explain the average behaviour within behavioural experiments through changes in selection strength and mutation rate, in distinction to Other-Regarding Preferences (ORP) models^17^,^18^,^19^,^20^,^21^ that aim to explain behaviour in terms of altruistic and envy parameters based on inequity aversion. The difference with the ORP models is that this stochastic model does not make any individual fairness or rationality assumptions: The main observation of Rand *et al.* using the UG as an illustration, was that when selection is not strong, meaning that the gains from the game do not strongly influence individual survival, allowing for chance to influence the evolutionary dynamics, the average behaviour of proposers and responders in the UG nicely matches the experimental observations^13^.

Here the stochastic evolutionary model is used to determine the range of parameter conditions that approximate our DG and AG results. However, even if the average behaviour can be nicely reproduced by the model for wide range of parameter settings, this stochastic evolutionary model ignores and fails to capture the strategic motivations of the dictators towards increased generosity. Moreover, the rather smooth and broad strategy distributions reproduced by the model do not match closely to the experimental ones. To overcome these issues we explicitly take into account the acceptance behaviour of the receivers in the AG as well as the impact of the dictators’ current decisions on future acceptance by newly matched receivers. By linking both experimental and theoretical results, we reveal here the real incentives behind dictators generosity, i.e. the strategic anticipation of ensuring a future interaction. In addition, the results show that reputation is essential to maintain the level of generosity, providing evidence for reputation as a mechanism that leads to “fair” outcomes as suggested by Nowak *et al.*^12^.

## Results

### Experimental results for DG and all AG treatments

**Figure 1:**
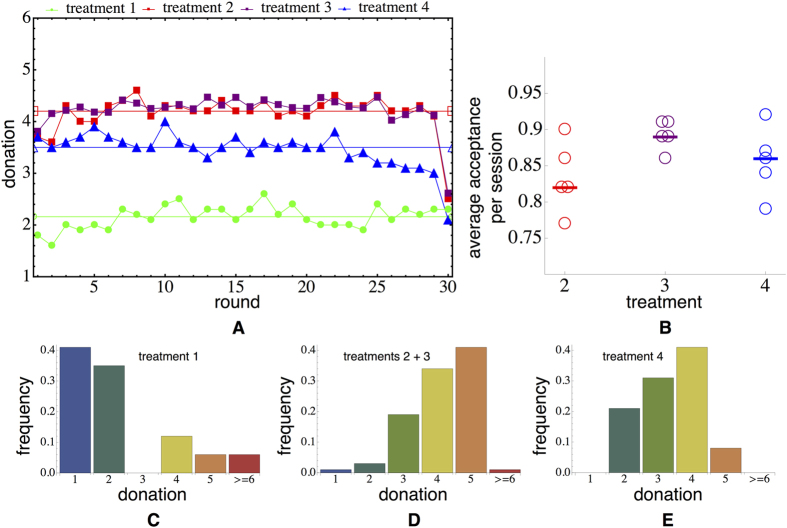
Experimental results for all treatments. (**A**) Donation levels vary depending on the treatment. In the treatment 1 (DG), the dictators give on average 2.2 ECUs. In the treatments 2 and 3 (AG), the amounts given reach on average 4.2 ECUs. Statistical testing revealed no significant differences for the donations between treatments 2 and 3. In the noisy AG, donations decrease to an average of 3.5 ECUs, which is significantly different from the earlier AG treatments (Welch two sample t-test on average donations per session, t = 3.7491, df = 7.982, p-value = 0.005655). (**B**) Receivers’ average acceptance rate per session. Even if there is no significant difference between the average acceptance levels of treatment 2 and treatment 4, there is a marginally significant difference in the acceptance levels of treatments 2 and 3 (Welch two-sample t-test on average acceptance per session: t = −2.4509, df = 5.369, p-value = 0.05449). The donation distributions (**C**) for treatment 1 (DG), (**D**) for treatment 2 and 3 (AG) and (**E**) for treatment 4 (noisy AG).

Although the subgame perfect equilibrium in a population of rational and selfish payoff maximizing individuals playing the DG is to give the minimum (positive) amount, the average donation within the treatment 1 (averaged over all 30 rounds) is close to 2.2 ECUs, which means that dictators keep on average less than 8 ECUs for themselves (see Fig. 1A). This deviation reveals the generosity of the dictators towards the receivers, highlighting the ORP^17^ intrinsic to the individuals involved in this experiment. Note that, as opposed to prior studies of the DG that the individual behaviour we observe is more varied than what has been seen in typical DG experiments (see Fig. 1C). This result may be due to the minimum of 1 ECU that has to be given by dictators in the experiment.

Switching to the AG clearly changes the amount given by the dictators (see treatments 2, 3 and 4 in Fig. 1A): The average amount given becomes ≈4.2 ECUs in the treatment 2 and 3 and ≈3.5 ECUs in treatment 4, deviating significantly from the subgame perfect equilibrium of the AG, which is equal to 1, as can be inferred by backward induction (Figure S1B). Moreover, receivers do not accept their partner in every matching, as visualised for all AG treatments in Fig. 1B. Hence, adding voluntary formation of couples to the DG leads to an increase in the average donations and introduces substantial levels of rejections, as was expected.

Although there is no significant difference between the amounts given in treatments 2 and 3, there is a small, but significant difference between the acceptance rates for those treatments, as can be observed in Fig. 1B. When adding the information on the number of times the matched dictator was refused by other receivers, the acceptance rate increases, on average, from 83% to 89%. The receivers’ acceptance/rejection rate is most likely due to a change in their expectations. One could hypothesise that the additional information in treatment 3 alters the receivers beliefs about their opponent in such a way that they are more likely to accept the match.

Treatment 4 shows clearly that when receivers are not continuously informed (and dictators are aware of this), the average donation decreases (see Fig. 1A). The exploitation of this situation by dictators becomes clear when comparing the donations in situations where the receiver was or was not informed about the past behaviour of the dictator (see Figure S5A in Supplementary Material). Our results reveal that there is again a significant difference in the amounts given by the dictators in both scenarios. Nonetheless, the amounts given in case the receiver was not informed do not drop completely to the level of the DG. The reason for this is due to the definition of the AG: As the amount given by the dictator in the uninformed case is still added to the history, dictators cannot give too little as such actions might be observed by another receiver in future interactions, potentially leading to a refusal to play the game. Interestingly, one can also observe that in the uninformed situation, receivers tend to accept more easily a dictator (see Figure S5B in Supplementary Material), yet acceptance is not 100%, which would be the rational thing to do.

As there is a clear difference between the amounts given in DG and AG, one can see the impact of this strategic motivation on the heterogeneity of the dictators’ behaviours. Figure 1C–E show, for all treatments, the dictators’ donation distributions. Whereas in treatment 1 the majority of the participants donate 1 or 2 ECUs to the receiver, the majority of the participants give 4 or 5 ECUs in the treatments 2 and 3, and 3 or 4 ECUs in treatment 4. This difference between the DG and AG is most likely the consequence of dictators’ concerns towards future encounters: As the amounts they give appear in their history they influence the probability of being accepted in the next rounds by another receiver. The difference between the DG and AG treatments reveals that dictators are willing to sacrifice a part of their payoff in order to create a favourable reputation. This generosity in the AG treatments is clearly strategic as the dictators give in the last round exactly the same as what they would give in the DG (see round 30 in Fig. 1A).

### Stochastic model parameters are predictive for the average behaviour in all games

Given these results for the DG and AG, we first examine for which parameter values of the standard stochastic evolutionary dynamics model^13^, i.e. the selection strength *β* and mutation rate *μ*, one obtains the closest fitting with the treatment results. As was argued, this stochastic evolutionary model allows to explain the outcome of a game by only considering how stochastic effects may lead to the behaviour observed in experiments.

We assume first that the behaviour of a dictator is defined by an integer value 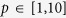 and the receiver’s behaviour by another integer value 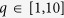. A dictator’s strategy simply specifies the amount she is willing to give to a receiver (in both DG and AG). A receiver’s strategy in the AG, which results in either the action “play” or “not play”, is conditional in the sense that *q* specifies the minimum amount expected in order to accept the matching with a given dictator. The receiver’s decision is based on the information provided after matching, i.e. the amount given in the previous rounds of the AG. For example, when a receiver with a threshold of expecting at least 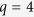 encounters a dictator that gave 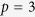 on average in the previous rounds, she will not accept the matching. We assume also that receivers have perfect information, meaning that they can always correctly infer the amount that would be given in the game by the dictator. In the context of the DG, a receiver’s strategy set contains only the “play” strategy, since she cannot make any decision to prevent the interaction.

Secondly, to capture also treatment 4, we introduce 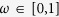, defining the likelihood that the receiver will know the last or average action (i.e. *p*) of her partner. Where 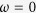 provides us with the results for the DG, 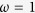 gives us the AG as used in treatment 2. As *q* defines the conditional strategy of the receiver in case they know the past donation *p* of the dictator, we only need to specify what they will do when no information is provided. For the sake of simplicity, we assume in the model that when no information is present each receiver expects no more than the average donation of the DG. As a consequence, the expectation of a receiver is a weighted combination of her original *q*, for the case when the dictator’s history is available (*ω*), and this baseline ECU when the history is not available 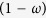. A detailed analysis concerning the motivation for this choice and different values for *ω* is provided in the Supplementary Material (see Figures S5 and S6).

Finally, to remain close to the experimental setup, players cannot change role. Consequently, the stochastic evolutionary dynamics model for the AG consists of two finite populations, one for the dictators and one for the receivers. We focus here on finite population evolutionary dynamics^22^,^23^, where each population contains *N* = 100 individuals and every game takes place between a randomly selected pair, one from each population (see Methods). In case of the DG, only the strategies of the dictators are relevant, thereby requiring only one population. Figure S3 in the Supplementary Material reveals in detail the stationary distribution and the fixation probabilities of the standard Dictator Game for the value of *β* that approximates the experimentally observed average donation of 2.2 ECUs, i.e. 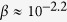.

**Figure 2:**
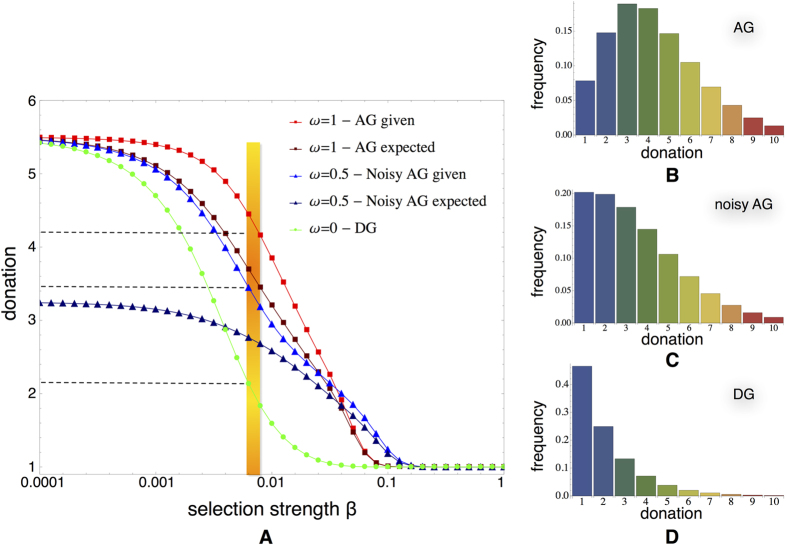
(**A**) Average donations given from the dictators and expected by the receivers with respect to the selection parameter *β* and the probability *ω* of knowing the dictator’s previous action according to the stochastic evolutionary dynamics model (low mutation rate 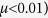. The dictators almost always give more when the approval of the matching lies on the receivers side, apart from when selection gets strong enough leading to the subgame perfect equilibrium outcome. Furthermore the latter, expect less than the amount they will finally receive (always 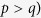. Black horizontal dashed lines correspond to the average donations observed in the experiments, around 4.2 in AG, 3.5 in noisy AG and 2.2 in DG, and the yellow background indicates this *β* area fitting them best. The distributions of the strategies of dictators are shown for the AG (panel (**B**)), its noisy variation (panel (**C**)), and finally the DG (panel (**D**)). A more detailed analysis of the DG strategy distribution can be seen in Figure S3 in Supplementary Material.

In Fig. 2A the average amounts given (*p*) in the DG, AG and noisy AG by dictators and the expected amounts by receivers (*q*) in the two AG models are visualised for varying selection strengths 
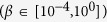
 and low mutation rate 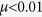 (see Methods). One can observe, when selection is very weak 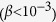 and thus evolutionary dynamics is mostly driven by neutral drift, that every donation level remains abundant with almost equal frequencies, resulting in average donations above 5 ECUs. Increasing selection strength 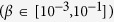 reduces *p* and *q* from more generous donations and higher expectations to the subgame perfect equilibrium in all games, which is maintained for all higher levels of selection strength 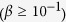. Similarly to the observations made for the UG^13^, the disadvantageous and advantageous inequity aversions are recovered for a wide range of stochastic evolutionary dynamics parameter values; in both DG and AG, dictators donate more than required and, in case of AG, receivers expect donations higher than the minimum. Mutation affects the results observed in Fig. 2A as in Rand *et al.*^13^ (see Figures S7 and S8 in Supplementary Material).

Comparing now the average donations for the DG and AG in Fig. 2A, one observes that the dictators’ anticipation increases the amount they give. The model reveals that in both games the same selection strength region holds for a given mutation probability , which is the region for 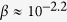. This result is quite intriguing as it provides novel insight into the predictive nature of the stochastic evolutionary dynamics model. That is, knowing the selection strength relevant in the DG provides information on the selection strength best fitting the average experimental donations in all AG treatments (and vice versa).

### Anticipating acceptance is key to success and generosity

Although the average behaviour matches nicely with the experiments, the strategy distributions generated by the stochastic evolutionary model do not reveal the same close fit , an issue that was also observed by Fowler and Christakis^14^. When comparing the heterogeneity in the experimental distributions in Fig. 1C–E to the distributions produced by the stochastic model (see Fig. 2B–D), one can observe that although the distributions for the DG and AG peak around the same average values, the generated distributions are much broader, including even many more donations above 5 ECUs than observed in the experiments. The results obtained for the noisy AG model with 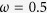 lead to even a worse correspondence; whereas the treatment 4 shows a peak around 3 and 4 ECU, the model provides results peaking around 1 and 2 ECU. This issue is a direct result of how the behaviours of the dictators and receivers are modelled: The amount a dictator gives depends here on how she thinks that amount will affect her chances to be accepted as a partner in the following rounds. Moreover, although receivers will refuse a partner with low previous donations, we might assume that they will not expect dictators to give up more than half of the endowment, which they themselves would also do if they would be a dictator.

**Figure 3:**
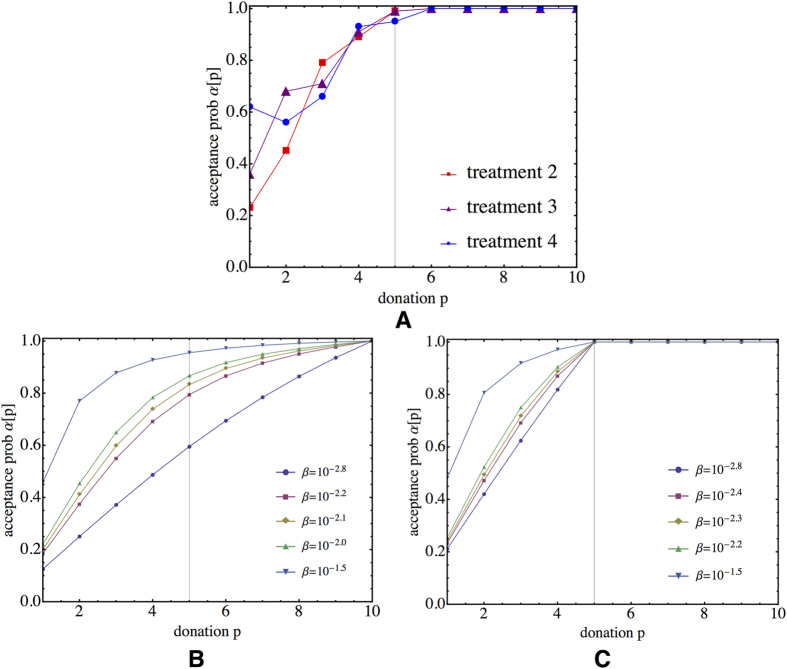
Acceptance probability in experiments and models. (**A**) The results of the AG treatments show that dictators will always be accepted in the next round once their average donation reaches the half of the endowment. (**B**) Acceptance probabilities generated by the basic stochastic evolutionary model with 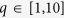 for different selection strengths *β*. (**C**) When 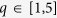, the probability of acceptance generated by the model always converges to 1 for 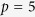, bringing the results closer to those observed in (**A**).

The latter assumption is supported by the experimental results (see Fig. 3A). The probability of acceptance, which we refer to as 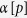, increases with the donation *p*. Donations bigger than or equal to 5 ECU are always accepted in all the three AG treatments. The current model with 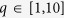 generates acceptance probabilities that are not as stringent: As can be seen in Fig. 3B, for different *β* approximating the average donations 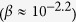, 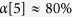. Moreover, even donations up to 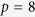 do not reach 100% acceptance. To address this issue, we restrict the expectation of the receivers to 5, i.e. 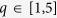, while keeping the strategies of dictators as before, i.e. 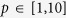. Under this modification, a receiver with 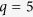 (the most demanding one) will accept to play with a dictator giving 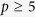, fitting the probability of acceptance on the experimentally observed results as can be seen in Fig. 3C.

This acceptance probability 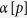 is also essential for the success of dictators: Dictators that consider how their donation influences the likelihood of being accepted in the future by another receiver have a strategic advantage over those that do not take this acceptance into account. The current evolutionary model ignores the importance of the future acceptance and therefore the future payoff by only using direct payoff to determine a dictator’s fitness. Following this reasoning we redefine a dictator’s success as a weighted combination of the current payoff 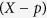 and the payoff potentially obtained in the next round 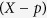 multiplied with the acceptance probability associated with the current donation *p*, resulting in the following dictator fitness function:


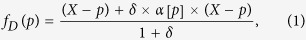


where *X* is the endowment amount and 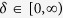 scales the importance of the future payoff in the overall fitness of the dictator. For 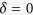 the future success is simply ignored, thus only the current payoff matters, recovering the basic stochastic evolutionary dynamics model discussed in the previous section. When 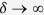, the success of the dictators will solely depend on their future payoff. Equation 1 is used when the interaction occurs (i.e. when 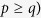. When 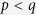, the receiver rejects the interaction, resulting in zero payoff for the present and the future.

**Figure 4:**
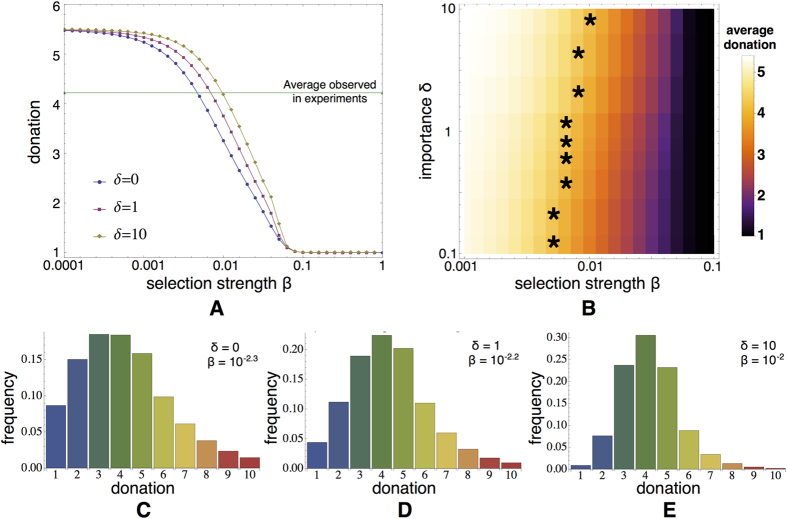
The role of future acceptance in fitting the experimental results. (**A**) Average dictators’ donation with respect to selection strength *β*. The more the future’s importance is taken into account the more we need to increase the intensity of selection *β* in order to fit our experimental data. (**B**) Experimental fit for a combination of the selection intensity *β* and the future importance factor *δ* (combinations annotated with the “*” represent the average donation observed in treatment 2). (**C**) The strategy distributions for the combinations of *β* and *δ* that fit best the average amount given we observed in treatment 2. Figure S9 in Supplementary Material shows similar results for the noisy AG, highlighting even more clearly the importance of acceptance and the payoff obtained in future interactions.

Results for the AG model with 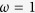 are visualised in Fig. 4. As can be observed in Fig. 4A for a given selection strength *β*, the more important the future payoff becomes (i.e. the higher the value of *δ*), the higher the average donation becomes. Or when comparing to the average behaviour observed in the AG experiment, the more important future acceptance becomes in the success of dictators, the stronger the selection strength needs to be to ensure the correspondence with the observations. This interplay between selection strength *β* and future importance *δ* is further explored in Fig. 4B. One can conclude that both *β* and *δ* parameters are important for tracking the average donations observed in the AG. Increasing the future importance *δ* leads to higher levels of intensity of selection *β*. Figure 4C–E show the stationary distributions of the dictators’ strategies for those configurations of {*β*, *δ*} that correspond more closely the average donation of 4.2 ECU observed in the AG treatments 2 and 3. For increasing *δ* the distributions in Fig. 4C–E become steeper around the average, revealing also a distribution of strategies closer to the AG experimental data: In case of 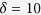, 80% of the population consists of individuals giving 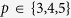 and also the likelihood of having donations higher than 5 is reduced significantly.

**Figure 5:**
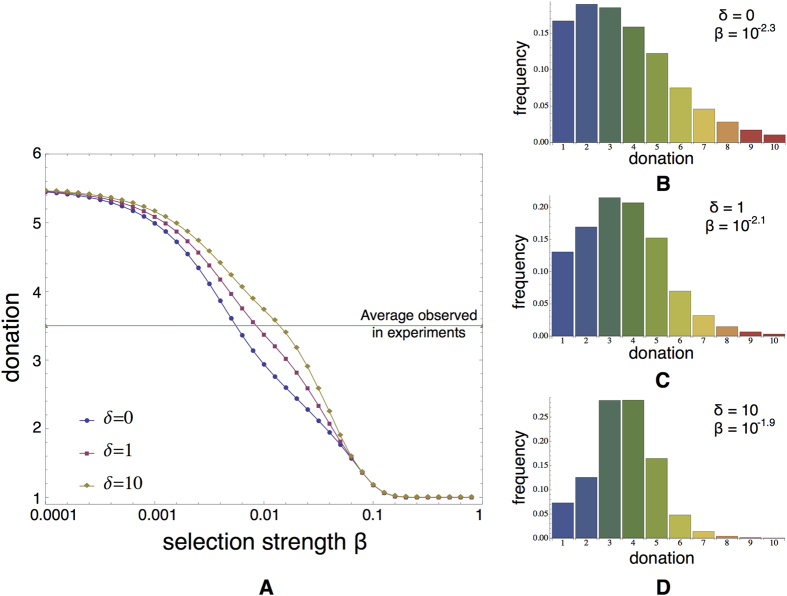
Effect of noise in the future payoff importance model. (**A**) The interplay of the intensity of selection *β* with the importance factor *δ* has the same effect in the average donations as in the AG. By increasing *δ* we witness more generous outcomes for a specific value of *β*. (**B–D**) Distribution of strategies for this *β* that fits best the experimental average, when future payoff importance is 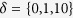.

These improvements remain valid and become even more clear when looking at the noisy AG where 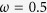. As before, the average donations are associated with the same selection strength (see Figure S10 in Supplementary Material), reinforcing again the predictive quality of the model for different games. More importantly, as is shown in Fig. 5, one can see that in order to obtain the distribution of donations close to what is observed in treatment 4 (see Fig. 1E), one should consider that 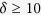 (which implies that 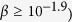: For these parameters values, the distribution also approximates the donations of 3 to 4 ECU. In other words, our analysis indicates that in our experiments dictators consider their future acceptance significantly more important than what they get immediately (on average). As such our extension to the stochastic evolutionary dynamics model provides an important improvement in explaining the results one can observe in our kind of experiments.

## Discussion

In this work we have presented the results of a novel behavioural experiment, which we call the Anticipation Game or AG, and an evolutionary analysis of those results to understand how acceptance by receivers influences the fitting of the stochastic evolutionary dynamics model to this experimental data. The AG introduces a voluntary matching phase wherein receivers decide based on the past behaviour of their matched dictator whether they want to play the DG. This new game, explicitly introduces a strategic concern in the decision process of the dictators; keeping more now will increase the likelihood that the future matched receiver will refuse to play the game.

Three treatments of the AG were performed, varying in the type and amount of information provided to the receiver. On one hand, the amounts given by the dictators are much higher than those given in the DG, and in both DG and AG more is given than the amounts theoretically predicted, under the assumptions of rationality and selfish payoff maximisation. On the other hand, even if rational and payoff maximizing receivers should always accept a dictator giving at least the minimum donation of 1 ECU, receivers’ acceptance levels never reach 100%. Adding more information (i.e. how many times a dictator was accepted or refused) did not induce changes in the donations, yet provided a marginally significant change in the acceptance by the receiver. Furthermore, introducing a 50% possibility that the receiver could not observe the past behaviour of the dictator, leads to a reduction in the average amount given, without any significant changes in the average acceptance level. Nonetheless, the presence or absence of the dictators’ history of donations in the decision making process produces significant differences in the average amount given and average amount expected within that fourth treatment, underlining again the importance of anticipation in the decisions made by dictators. These results provide additional evidence that reputation is essential in a partner selection mechanism, as was argued from different perspective by Nowak *et al.*^12^.

Using stochastic evolutionary dynamics wherein dictators give a fixed amount and receivers decide whether to play conditionally, we observe that the model parameters are predictive for all our four treatments. In all cases, the same weak selection (*β*) region is retrieved, with dictators always giving more than expected 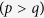 and receivers expecting more than the minimal amount 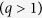. We show hence that the predictions provided by the stochastic model are robust for different variations of the game. This remarkable observation is in line with the recent experimental findings, suggesting that there may exist a cooperative phenotype, i.e. an individual’s behaviour in one cooperative context is related to his or her behaviour in other settings^24^,^25^,^26^. Nonetheless the heterogeneity in the strategies actions observed in the experiments is not recovered by the model.

We propose here an important extension to the model so as to improve the correlation between the distributions of strategies generated by the model and those observed in the different AG treatments. We explicitly model the anticipation of the dictators with respect to the expectations of the receivers by a system-level parameter *δ*, which determines how important the payoff one may obtain in the next round is for the current success of the dictators. Our results show that increasing *δ* alters the selection strength *β* that comes closest to the experimental data (and vice versa): The more the future is taken into account the more the selection strength needs to increase in order to match the data. Furthermore, the acceptance probabilities following from the model correspond much better with what we can observe in the experiments and the predictive properties of the earlier model are maintained in the extended model, which is supported here by the results obtained for the AG and noisy AG (see Figure S10 in Supplementary Material). As such, both the probability of acceptance and the *δ* parameter play an essential role when trying to match the stochastic evolutionary dynamics model to games that include acceptance and future gain concerns.

Note that although we have here aimed to understand the behaviours of the dictators and receivers from an evolutionary perspective, this does not preclude the use of other game theory models to analyse the experimental data. Game theory literature has a large history of models that could be used to address the question on how the behaviour came about^9^. An exhaustive analysis of the most useful model is beyond the scope of the current manuscript.

Our AG experimental results capture findings similar to what one can expect in the UG^12^,^18^,^27^, yet, whereas the concerns of the dictator in our AG are associated with future interactions, the concerns in the UG are linked to the game itself. Moreover, there is no voluntary matching occurring in the UG, as both players are expected to play the game even when they might not be interested in their co-player. Notwithstanding these differences, the conclusions we draw here might also be extendable to the UG. Future work will indicate whether indeed a parameter similar to *δ*, which represents the strategic concern of the proposer, will also bring the stationary distributions of the proposers in the UG closer to the distributions of offers observed in UG experiments. It will be now interesting to see whether variations of the AG or UG experiment can be defined that would allow us to determine the average *δ* and its distribution from experiments. In addition, models should be designed wherein the parameter *δ* becomes part of the behaviour of the individuals as opposed to being a system-level parameter, exploring the conditions that allow individuals that take the future into account to evolve. All these issues go beyond the current manuscript and are being analysed in subsequent research.

Since receivers use the reputation of dictators to decide whether to play or not, this research is reminiscent of the evolution of cooperation in systems based on indirect reciprocity^28^,^29^,^30^. In those systems individuals acquire a reputation based on how they treat other individuals, where a good reputation may lead to future benefits provided by other individuals. Evolutionary models have shown that individuals’ reputation effects may lead to more fair outcomes^31^. Nonetheless the current AG work differs from indirect reciprocity models, since here the reputation is implicitly determined by the one that directly benefits from it: In indirect reciprocity, the reputation is assigned by other players that observe how one individual treats the other^32^ whereas here, an individual determines their reputation directly by accepting a loss now in order to guarantee benefits in the future.

Our work is therefore closer to recent research on competitive altruism^5^,^33^,^34^. Competitive altruism is based on three assumptions; 1) individuals should differ in strategy or resources, 2) individuals have access to reliable information on the reputation or past actions of their co-players, providing a reliable guide to future behaviour and 3) individuals are paired in an assortative manner, meaning that the highest contributors have privileged access to the most profitable partners in the partner choice process. As was argued in the introduction the latter feature is also implicit to biological market models^1^,^2^,^3^,^6^. Such a preference-based matching is absent in AG as receivers decide to accept a randomly proposed dictator. As such our results show that even without having priviledged access to the most cooperative individuals, thus without an explicit competition between the dictators for partners, generous donations emerge.

Our stochastic evolutionary model also does not require demography differences or market effects^6^ to explain the generosity observed in the experimental work. In order to explain dictators’ generosity in the AG it is necessary to take into account the influence of the receivers’ other regarding preferences as well as the dictators’ concern about the possibility of being accepted by the receivers. Hence even in a world dictated by stochastic effects, strategic considerations need to be incorporated to provide ultimate explanations for generous outcomes observed in experiments with human participants.

## Methods

### Experiment setup

We performed the DG and AG experiments at the CentERlab of the University of Tilburg, in the Netherlands. Five sessions were performed for the treatments 2, 3 and 4 (all AG variations). For treatment 1 we conducted two DG sessions with identical action choices for the dictators as in the AG to ensure a clear comparison. All 292 participants were students of the University of Tilburg, excluding Economy students. There were 148 male and 144 female participants with an average age of 23.3 years. On average 18 subjects participated per session, earning between 7 to 14 Euros. At the beginning of each session, the participants were randomly assigned the role of dictator or receiver, which they maintained for the entire session. Then, after drawing a number and being seated to the specified computer accordingly, the instructions of the session were read aloud where we made it explicitly clear that the whole experimental process lasts for 30 rounds. Afterwards and before playing the game, the participants performed a test to ensure that they understood the game. At the start of the AG sessions (initiation phase), the subjects first played three rounds of the standard DG, creating an initial history or reputation for each dictator. The partner choice phase is then used in the following 27 rounds of the AG. Using the reputation information (in case of treatment 2), the receivers decide whether to play or not with a given dictator. If the game is not played, they both receive zero payoff and need to wait for the next random matching. When playing, the dictators receive an endowment of 10 ECUs and they need to decide how much (between 1 ECU and 10 ECUs) they will give to the receiver. Once a part of the endowment is transferred to the receiver this amount is added to the history of that dictator, replacing the oldest value in the list. This update only occurs when the game has been played; otherwise the three-round history remains the same. At the end of the session the ECUs gathered by each individual were transformed in a monetary gain. A more detailed description of the experimental procedures is provided in Supplementary Material. All experimental data is available upon request from the corresponding author.

### Stochastic evolutionary dynamics model

We analyse the evolutionary dynamics of individual strategies in populations of finite size *N*^22^,^35^,^36^,^37^. A mix of neutral drift, which accounts for stochastic effects, and natural selection, dictates the dynamics in this model. The balance between these two forces is controlled by two parameters: the intensity of selection *β* and mutation rate *μ*. In a stochastic process strategies with low fitness will have a small yet non-zero chance to survive.

Although different social dynamics are possible, we assume here that the individual strategies spread through the pairwise comparison rule, according to which two individuals A and B are randomly drawn from the population at each time step, and the player with strategy A imitates the one playing strategy B with probability given by the Fermi distribution function^38^,^39^:


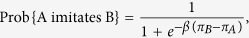


where 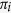 denotes the average payoff of player with strategy *i* after interacting once with every other individual in the population. Parameter *β* denotes the intensity of selection, tuning how much the payoff contributes to the fitness of a player. In the limit 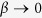, selection does not play any role; neutral drift drives the dynamics, i.e. both strategies will have prevailing probabilities proportional to their abundance. On the contrary, when 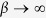, evolution proceeds deterministically, as the individual with the lower payoff will adopt the other individual’s strategy regardless of how large the difference is.

Assuming this pairwise comparison rule, a Markov chain model can be constructed to describe the evolutionary dynamics of A and B strategies in a finite population. The different configurations of the population define the states of the Markov chain, ranging from a state with only A strategists to a state with only B strategists. The transition probability between any two states indicates the probability that the population moves from one state to the other in one time step. We distinguish between two kinds of states, the absorbing or homogeneous ones where the population composes of either strategy A or strategy B solely, and the transition ones, including the rest of the population compositions. The stochastic nature of the dynamics ensures that one of the strategies always survives, i.e. the Markov process will end up in either of the two absorbing states.

More specifically, suppose there are *k* individuals playing strategy A 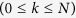 and 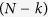 individuals playing strategy B. The (average) payoff of the individuals that uses A and B can be written respectively as:


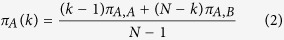



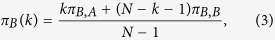


where 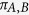 stands for the payoff an individual using strategy A obtains in an interaction with another individual using strategy B. The probability to increase (or decrease) the number *k* of individuals using strategy A by one in each time step can be written as:


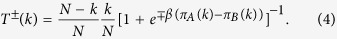


The probability that a single mutant A will take over the whole population of individuals adopting strategy B, dubbed the fixation probability of strategy A against B, is given by^22^,^23^,^35^:


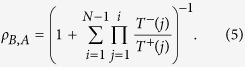


Considering the Markov process through millions of time steps, mutants may appear and disappear or even prevail in the population. The process shall move from one homogeneous state to another following the different fixation probabilities, assuming that mutations are rare , which in our case corresponds to mutation rates 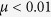. Taking into account a set 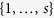 of different strategies, these fixation probabilities determine a transition matrix 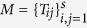, with 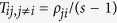 and 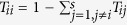, of a Markov Chain. The normalised eigenvector associated with the eigenvalue 1 of the transposed of M provides the stationary distribution^22^,^23^,^35^, which shows how many times a Markov process will reach a homogeneous state or even which is the probability the process will end up in a specific homogeneous state after infinite rounds or time steps.

In AG, we study evolutionary dynamics in bipartite populations, i.e. one population of dictators and one population of receivers. Individuals interact between populations, yet evolve within their own. More specifically, a homogeneous state corresponds to a pair of strategies, one for dictators and one for receivers. Suppose that we are in a homogeneous state and then a mutant A appears in only one of the populations, for example the dictators’ population with residents playing B. We do not consider then another mutant in the receivers’ population before the previous one either gets fixated or eliminated. Therefore, fixation probability of A depends also on the state of the other population (Figure S4 in Supplementary Material). As a consequence, a transition between two homogeneous states presupposes that the strategy in one of the populations remains the same.

To determine the region of *β*-values that produce the model results (i.e. stationary distributions of *p* and *q* values for dictators and receivers respectively) close to those we observed in the experiments, one simply increases the *β*-value, assuming a small enough step-size, until an average *p* (or average *q*) is reached that becomes smaller than the experimental value. This approach is valid since these average values are decreasing functions of *β* (see Fig. 2). This *β* together with the prior provides roughly the region for which the model matches the experimental data.

## Additional Information

**How to cite this article**: Zisis, I. *et al.* Generosity motivated by acceptance - evolutionary analysis of an anticipation game. *Sci. Rep.* **5**, 18076; doi: 10.1038/srep18076 (2015).

## Supplementary Material

Supplementary Information
