# Supplementary material for: Generosity motivated by acceptance - evolutionary analysis of an anticipation game
Source: Sci Rep. 2015 Dec 14;5:18076. doi: 10.1038/srep18076 (PMC4677303; doi:10.1038/srep18076)
Supplement: Supplementary Information [file srep18076-s1.pdf]

# Supplementary Material: Generosity motivated by acceptance - evolutionary analysis of an anticipation game


Ioannis Zisis[α], Sibilla Di Guida[β], The Anh Han[γ],

Georg Kirchsteiger[δ,ε] and Tom Lenaerts[α,ζ,*]

[α] MLG, Département d'Informatique, Université Libre de Bruxelles, Brussels, Belgium

[β] Department of Business and Economics, COHERE, Syddansk Universitet, Odense, Denmark

[γ] School of Computing, Teesside University, Middlesbrough, UK

[δ] ECARES, SBS-EM, Université Libre de Bruxelles, Brussels, Belgium

[ε] CEPR, CESifo and VCEE

[ζ] AI lab, Computer Science Department, Vrije Universiteit Brussel, Brussels, Belgium

[*] Corresponding author: Tom.Lenaerts@ulb.ac.be

# Contents

**1 Defining the games** **3**

**2 Details concerning the experiments** **3**

**3 Statistical differences in treatment 4 between situations with and without reputations** **8**

**4 Receivers' expectations in the noisy AG when there is no information** **10**

**5 The effect of mutations** **12**

5.1 Agent-based simulations . . . . . . . . . . . . . . . . . . . . . . . . . . . . . 12

5.2 Local mutation mechanism . . . . . . . . . . . . . . . . . . . . . . . . . . . . 14

5.3 Mutation under weak selection dynamics . . . . . . . . . . . . . . . . . . . . . 15

**6 Extended stochastic model for the noisy AG** **17**

# 1 Defining the games

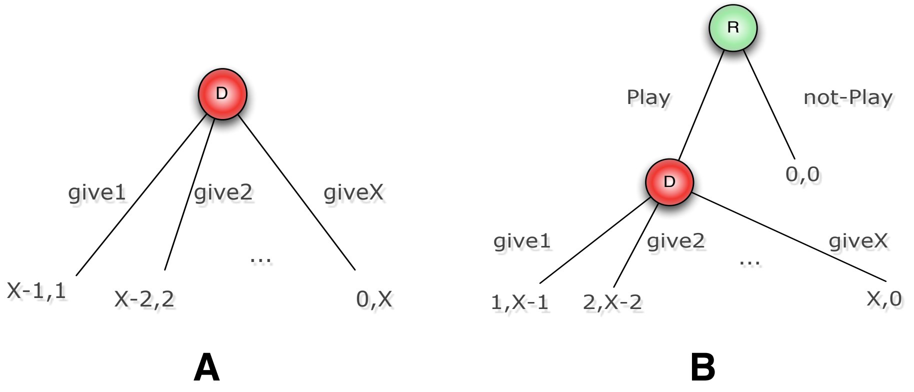


**Figure S1.** The games under study, the Dictator Game (DG) and the Anticipation Game (AG). Initial endowment is $X$.

The subgame perfect equilibria for DG i.e. $\{$give1$\}$, and AG, i.e. $\{$Play, give1$\}$, can be obtained through backward induction. Note that if the minimum donation had been $0$, then the receiver in the AG would have been indifferent between playing and not playing, as in both cases she would obtain the same payoff, which is zero. To avoid any confusion in the experiments, we decided therefore to make the minimum donation equal to $1$, for both games.

# 2 Details concerning the experiments

All experiments were performed in the CentERlab at the University of Tilburg in the Netherlands. The participants of our experiments were all students of the University of Tilburg, excluding students with an Economic background as they may be familiar with game theoretic concepts. All experiments were performed using z-Tree [1].

The first experiments, consisting of treatments 1 to 3, were performed in February 2013. For treatment 1 we only had 2 sessions for the DG, as the existing literature (see Chapter 2

of [2]) provides already substantial information on this type of game . In treatment 2, 5 sessions of the Anticipation Game, where receivers know the past three donations of their matched dictators, were performed. And similarly, 5 sessions were conducted for treatment 3, wherein the receivers were given the extra information of how many rounds their partner got rejected in her previous interactions.

At the beginning of each session, the subjects drew a number, specifying the position they take in the computer room. Each computer had a predefined role of either dictator or receiver, which we annotated as Agent 1 or Agent 2 respectively. The instructions of the session were read aloud by one of the experimenters and the participants were asked to complete a small questionnaire to check whether they understood the experiment. For treatment 2 the following text was read aloud (similar texts were used for the other treatments):

> Dear Participant, welcome!
>
> You are about to participate in an experiment on interactive decision-making, conducted by researchers from the Vrije Universiteit Brussel and the Université Libre de Bruxelles, and funded by the Belgian fund for the scientific research (Fonds de la Recherche Scientifique). In this experiment you will earn some money, and the amount will be determined by your choices and by the choices of the other participants.
>
> Your privacy is guaranteed: results will be used anonymously.
>
> It is very important that you remain silent during the whole experiment, and that you never communicate with the other participants, neither verbally, nor in any other way. For any doubts or problems you may have, please just raise your hand

and an experimenter will approach you. If you do not remain silent or if you behave in any way that could potentially disturb the experiment, you will be asked to leave the laboratory, and you will not be paid.

All your earnings during the experiment will be expressed in Experimental Currency Units (ECUs), which will be transformed into Euros with a change rate of 30 to 1. At the end of the experiment, a show up fee of 5 euros will be added to your earnings.

You will be paid privately and in cash. Other participants will not be informed about your earnings.

Before starting, you will be randomly assigned to the role of Agent 1 or Agent 2, and you will maintain the role for the whole experiment. Agents 1 and 2 will form pairs of one Agent 1 and one Agent 2 each.
The experiment is divided in two parts, for a total of 30 rounds. In each round there will be a random re-pairing of Agents 1 and 2. Obviously, as the matching rule is random and as the number of rounds is larger than the number of participants, during the experiment you will be paired more than once with the same subject. However, you will never know the identity of the participant you are matched with and hence you will not be able to identify your partner.

PART 1

The first part of the experiment consists of 3 rounds. In each round each Agent 1 receives an endowment of 10 ECUs and has to decide how much to give to the Agent 2 that has been matched with him/her. The minimal amount given to Agent 2 is 1 ECU, the maximal 10 ECUs. After the choice, each Agent 2 will be informed

about the amount that has been given to him/her.

In this part of the experiment, Agents 2 cannot directly interact with Agents 1, but simply observe.

PART 2

The second part of the experiment consists of 27 rounds (from round 4 to round 30). At the beginning of each round a screenshot will show to each Agent 2 what the randomly matched Agent 1 gave in the three previous played rounds. Agent 2 will then have to choose whether he/she intends to interact with that specific Agent 1 or not.

**IF NOT** - Agent 2 refuses to interact and both Agent 1 and 2 skip the round, going directly to the following one, where they will be matched with new partners. When an interaction is refused, both Agent 1 and 2 gain 0 ECUs for that round. Refusals are not shown in the screenshot that summarizes the three previous periods.

**IF YES** - Agent 2 accepts to interact. Agent 1 receives 10 ECUs and chooses how much to give to Agent 2, with a minimum of 1 and a maximum of 10 ECUs. After the choice, each Agent 2 will be informed about the amount that has been given to him/her.

As said, at the beginning of each round a screenshot will present to each Agent 2 what the randomly matched Agent 1 offered in the three previous played rounds. Agent 2 therefore will not see if in the previous rounds other Agents 2 refused to interact with that specific Agent 1.

Once the experiment is over, you will have to fill a short questionnaire.

After that, your final earnings will be determined. For Agent 1 the final earnings (in ECUs) are the sum of all those amounts he/she did not give to his/her Agent 2 in those rounds where he/she was accepted by Agent 2. For Agent 2 the final earnings are the sum of all those earnings he/she receives from his/her Agent 1 during the rounds in which he/she did not refused to interact with Agent 1.

These final earnings are transformed into Euros with 30 ECUs being equal to 1 euro.

Your final earning will appear on the screen and the experimenters will explain the modality of payment.

Thank you for your participation!

After reading this text and filling in the questionnaire, the session started. In Figure S2, one can observe two of the game screenshots, one for Agent 1 and the other for Agent 2.

At the end of the experiment, the participants had to reply to a small questionnaire regarding either their motives of the level of donations they gave (for Agents 1) or the reasons they accepted or rejected a certain dictator (for Agents 2).

Once finished they were then asked to pass by the payment desk to receive their monetary gains. On average 18 subjects participated per session, and each participant earned an amount of money ranging from 7 to 14 Euros. The same procedure was used for all three treatments.

The fourth treatment for the noisy AG was performed in November 2013, excluding subjects that already participated in the earlier treatments. Average participation was 16 subjects per session, earning an amount of money ranging also from 7 to 14 euros. The procedure followed was the same as the one described before.

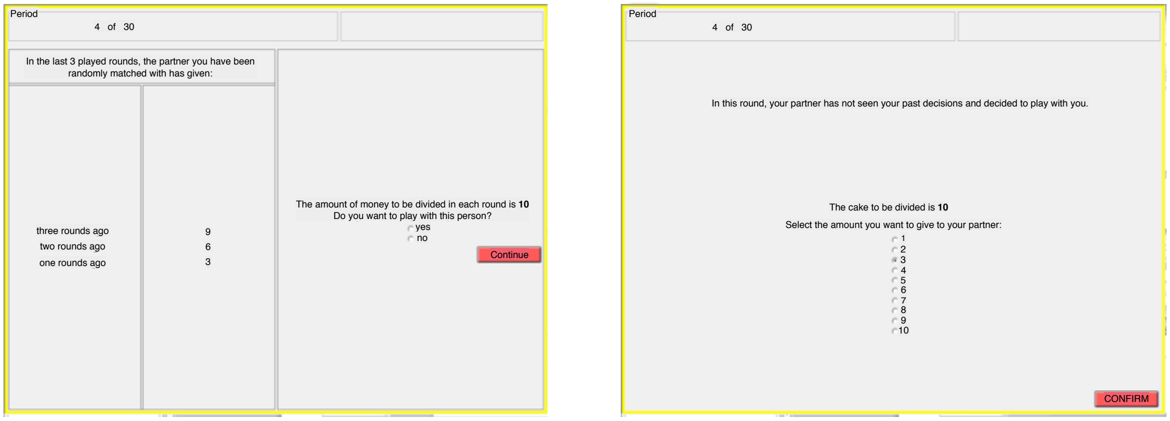


A B

**Figure S2.** Two example screenshots used during the experimental session. **A**. A certain receiver (Agent 2) has to decide whether to accept to play with a certain dictator after observing her 3 previous donations. This screenshot was taken from treatment 2 (AG). **B**. A dictator (Agent 1) in treatment 4 (noisy AG) has to decide how much to give to her partner after being informed that the latter has not observed her 3 previous donations.

# 3 Statistical differences in treatment 4 between situations with and without reputations

As briefly described in the main text, we observed differences in the amounts given by the dictators and the acceptance rates by the receivers when the latter could observe (or not) the previous three donations of their matched dictator. Analysing the results in Figure S5 shows that there is a significant difference in the donations (Welch two sample t-test, t=-3.05, df=4 and p-value = 0.03693) and the acceptance rates (Welch two-sample t-test, t=-3.6685, df=4 and p-value = 0.02142) of the noisy AG in the case where there is a history or not. Given the almost $95\%$ of acceptance rate when receivers cannot observe the dictators' history (as shown in Figure S5B), one might assume in the stochastic modelling that a receiver will almost always accept an interaction with a dictator when she does not know how much the dictator gave in previous

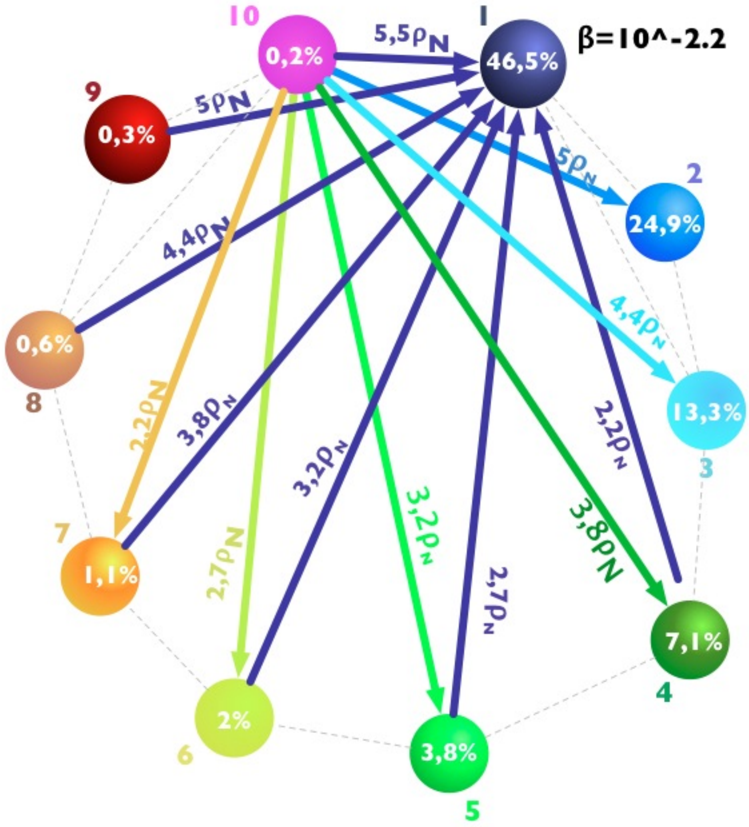


**Figure S3.** Stationary distribution and fixation probabilities in the Dictator Game where each state corresponds to the amount given from dictators. Even if the most common strategy is the selfish one of giving 1, there is still a 25% of donating 2 and another 25% allocating from 3 to 5, the latter may be considered as fair players. This distribution matches nicely with the one we found in our experiments (Figure 1C in the main text). The arrows refer to the fixation probability of the strategy at the end of them. Dotted lines between states correspond to neutral drift. Only the transition dynamics referring to state 1 and 10 are shown. All the rest can be deduced from them, e.g. from state 9 to 6 selection favors the latter with fixation probability of $2.2\rho_N$, same probability with the one from state 10 to 7. $N = 100$ and $\beta = 10^{-2.2}$.

rounds.

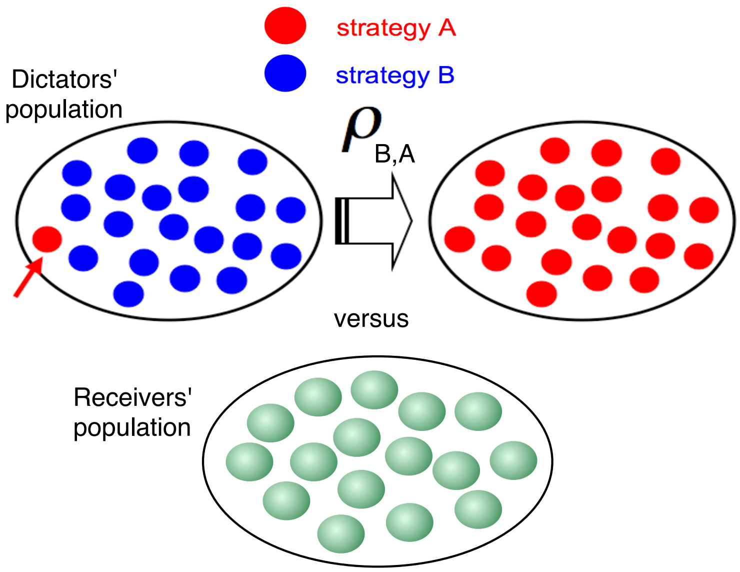


**Figure S4.** Fixation probability $\rho_{B,A}$ of a single mutant arriving in dictators' population. The mutant plays strategy A when entering in a population of agents playing strategy B. We do not allow for mutants in receivers' population; thus their strategy remains steady. Then $\rho_{B,A}$ is the probability the A mutant will prevail within the dictators' population while interacting with receivers belonging to the receivers' population.

# 4 Receivers' expectations in the noisy AG when there is no information

As can be observed in Figure S6, for the intermediate selection strength of $\beta = 10^{-2.2}$, increasing $\omega$ boosts generosity and therefore dictators' donations, or putting it differently the less receivers know regarding their matched dictators' behavior, the lower the donations we observe. As has already been described in the main text, for $\omega = 0$ and $\omega = 1$, the donations coincide with the DG and AG respectively, i.e. around 2.2 and 4.2 ECUs respectively. However, in determining the average amount expected by the receivers, one needs to take into account that the expectations defined by $q$ for each receiver need to be modified when the history information

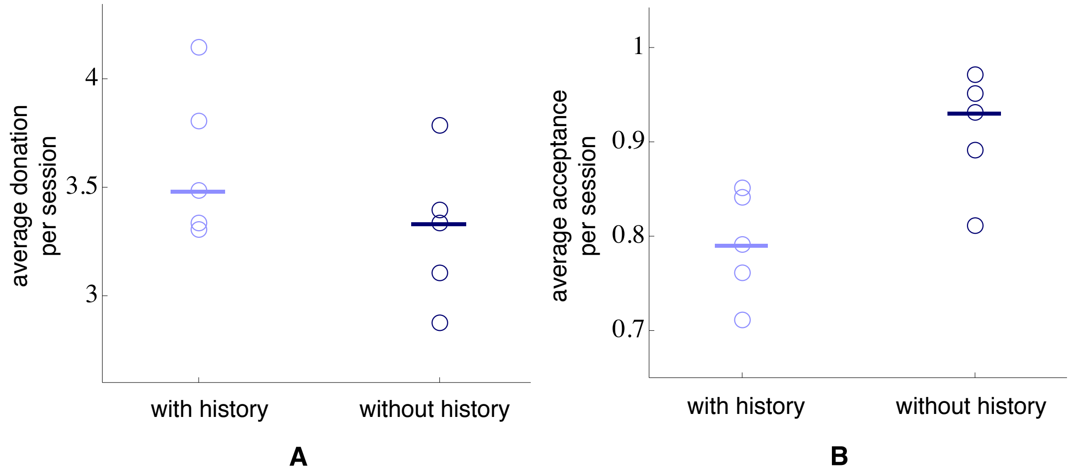


**Figure S5.** Donation levels and acceptance rate per session for the fourth treatment, the noisy AG, when receivers obtain or not their matched dictators' history. Initial endowment is $X = 10$.

is absent. Clearly, receivers have different expectations when they obtain and do not obtain information about their matched dictator. Figure S6, shows that if one does not adjust the expectation, the receivers seem to expect more for decreasing values of $\omega$, leading to the loss of one of the inequity aversions: the amount given becomes less than what is expected, which does not make much sense in light of the available data. If we assume in the model that the expectations are different when information about the past behaviour of the dictator is present or not, then we can recover $p > q$. One can assume for instance that the receivers expect the same as in the DG, which is either $1$ ECU or the $2.2$ ECUs as we have observed experimentally. Then we make a weighted combination of the expectation, given by $q$, for the case when the dictator's history is available ($\omega$) and $1$ ECU (or $2.2$ ECU) when the history is not available ($1 - \omega$). We observe in Figure S6 that by altering the expectation to $1$ ECU in the model when the history is not available guarantees again that $p > q$. Moreover, the expectation of the receivers nicely tunes between the extremes $1$ ECU and $3.7$ ECU provided by the DG and AG respectively.

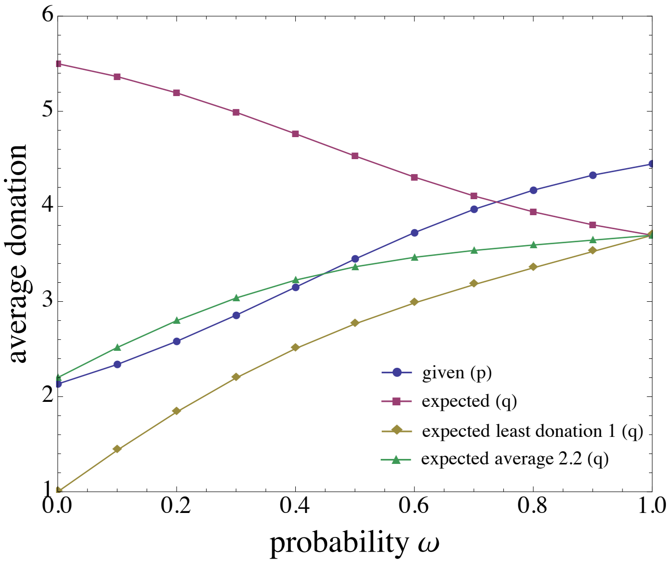


**Figure S6.** The effect of $\omega$ on the average donations given or expected when $\beta = 10^{-2.2}$. The dictators may take advantage of the fact that the receivers are less informed (i.e. lower $\omega$) in order to donate lower amounts. We depict here 3 different expectation threshold lines for the receivers with respect to which donation they expect to receive when they do not obtain the history of their partners.

# 5 The effect of mutations

## 5.1 Agent-based simulations

In our agent-based computer simulations we defined two distinct populations of size $N = 100$, one for the dictators and one for the receivers. A random strategy is assigned to each individual at the beginning of the simulation. We assume 10 different strategies in each population. For the dictators this corresponds to the 10 different amounts given $\{1, 2, 3, 4, 5, 6, 7, 8, 9, 10\}$ and for the receivers to their expectance thresholds, i.e. a receiver with strategy 5 will accept to play with dictators that give at least 5 meaning the ones that have the strategy of giving $\{5, 6, 7, 8, 9, 10\}$.

In each generation a random matching is proposed and they all play the game. Then, ac-

cording to the pairwise imitation rule, imitation takes place (with probability $1 - \mu$) in one of the populations only. Two agents i, j are randomly picked and then agent i imitates the agent j's strategy with probability $\frac{1}{1+e^{-\beta(\pi_j - \pi_i)}}$, which corresponds to the Fermi distribution function [7]. When both populations approximate fixation (meaning at least 90% fixated), then the corresponding pair of strategies counter gets raised by 1. Moreover with a certain probability $\mu$ (mutation rate parameter) one agent's strategy from one population will change randomly. Each simulation is run for $10^9$ generations.

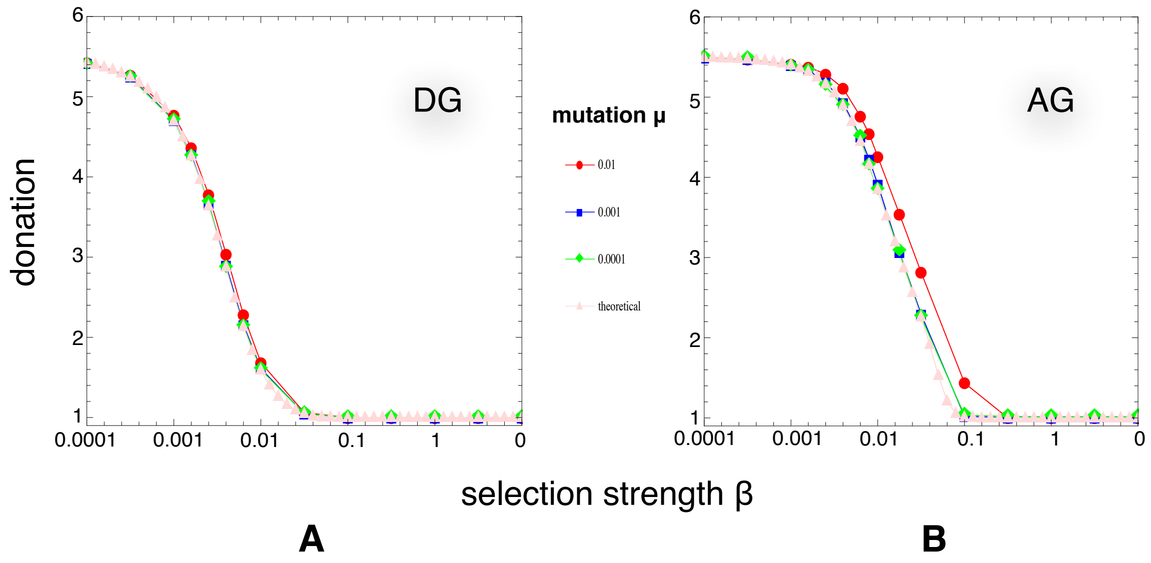


**Figure S7.** Average donations given by the dictators in function of the selection parameter $\beta$ and a specific mutation rate $\mu$. One can notice a slight increase in average donations when the mutation rate is increased, as was also observed in [9] .

We count how many times a certain pair of strategies fixates, and from that, the frequency of a pair is determined, giving the stationary distribution. Combining the distributions with the agents' strategies, one can compute the average amount given and average amount expected for dictators and receivers respectively. Each simulation ($10^9$ generations) for a pair of $\beta$ and $\mu$ is repeated for 100 times and averaged afterwards to obtain the final result. Values for mutation parameter $\mu$ were $\{0.01, 0.001, 0.0001\}$. For instance, for $\mu$ is 0.01, we have, on average, one

mutation per 100 generations, but only in one of the populations. Results are shown in Figure S7.

## 5.2 Local mutation mechanism

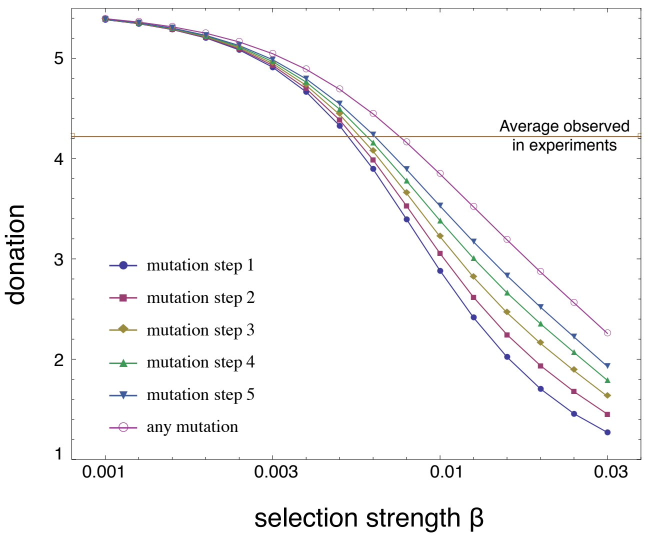


**Figure S8.** Average donations given from the dictators with respect to intermediate levels of the selection parameter $\beta$ when we vary the mutation step. The higher the mutation step, the larger the range of strategies a mutant may adopt and then randomness gets increased.

We also considered variations in the way mutations may occur. Whereas the earlier form (see Figure S7) is global, one can also consider that a strategy can only change to a ”similar” strategy. We define here the mutation step as the range of strategies a certain agent may adopt around her current strategy. The larger the mutation step the more strategies a mutant may switch too. For example, when the mutation step is 1, a dictator playing the strategy 3 can only mutate to strategies $\{2, 3, 4\}$ and not the rest. From the receivers' side, e.g. when mutation step equals to 3, a mutant of the strategy 2 can only mutate to strategies $\{1, 2, 3, 4, 5\}$. The results in Figure S8 show the effect of this kind of mutation on the average donations for varying the mutation step and $\beta$. As can be observed: changing the mutation step has an effect on the

selection strength $\beta$ best fitting the experimental data. The higher the randomness (from step size 1 to 5, until global mutation), the higher the $\beta$ needs to be in order to match the experimental results.

## 5.3 Mutation under weak selection dynamics

Suppose a player from the dictators' population has a strategy of donating a fixed amount $p \in [0, 1]$ when playing, and a proposer form the receivers' population has a strategy of accepting to play when the expected amount from the paired dictator is at least $q \in [0, 1]$. Furthermore, assuming that with a probability $s \in [0, 1]$ the receiver does not have prior information about the paired dictator. In that case, we consider that the receiver always accepts to play (as it is the rational choice). This probability $s$ corresponds to the $(1 - \omega)$ as defined in the main text.

Hence, when these two players meet, their payoffs are given by:

$$A_{dic} = \begin{cases} 1 - p, & \text{if } p \geq q \\ s * (1 - p), & \text{otherwise.} \end{cases} \tag{1}$$

$$A_{rec} = \begin{cases} p, & \text{if } p \geq q \\ s * p, & \text{otherwise.} \end{cases} \tag{2}$$

The parameters: $N$: population size; $\mu$: mutation rate.

Similarly to Rand [9], we discretize the problem: $p = i/m$ and $q = j/m$, where $m \geq 1$ is an integer and $1 \leq i, j \leq m$. As shown from Otshuki [10], for weak selection, the combination of dictators' and receivers' strategies that is most favoured by selection (i.e. most abundant) is

the one that maximizes $L(i/m, j/m) + 2(N-1)\mu.H(i/m, j/m)$, where:

$$L(\frac{i}{m}, \frac{j}{m}) = \frac{1}{m^2}\sum_{i',j=1}^{m}[A_{dic}(\frac{i}{m}, \frac{j}{m}) - A_{dic}(\frac{i'}{m}, \frac{j}{m}) + A_{dic}(\frac{i}{m}, \frac{j'}{m}) - A_{dic}(\frac{i'}{m}, \frac{j'}{m})]$$
$$+\frac{1}{m^2}\sum_{i',j=1}^{m}[A_{rec}(\frac{i}{m}, \frac{j}{m}) - A_{rec}(\frac{i}{m}, \frac{j'}{m}) + A_{rec}(\frac{i'}{m}, \frac{j}{m}) - A_{rec}(\frac{i'}{m}, \frac{j'}{m})]$$
$$H(\frac{i}{m}, \frac{j}{m}) = \frac{1}{m^2}\sum_{i',j=1}^{m}[A_{dic}(\frac{i}{m}, \frac{j}{m}) - A_{dic}(\frac{i}{m}, \frac{j'}{m}) + A_{rec}(\frac{i'}{m}, \frac{j}{m}) - A_{rec}(\frac{i'}{m}, \frac{j'}{m})]$$

Simplifying the equations we obtain:

$$L(\frac{i}{m}, \frac{j}{m}) = IS(i,j) - \frac{2i^2(1-s)}{m^2} - \frac{j^2(1-s)}{m^2} + \frac{i(1-3s)}{m} + \frac{j(1+m)(1-s)}{m^2} - \frac{(1+m)(1-3s)}{2m}$$

$$H(\frac{i}{m}, \frac{j}{m}) = -\frac{i^2(1-s)}{m^2} - \frac{j^2(1-s)}{2m^2} + \frac{i(1-2s)}{m} + \frac{j(1-s)}{2m^2} + \frac{(1+m)s}{2m}$$

where $IS(i \geq j) = 1-s$, if $i \geq j$, and 0 otherwise. Now let $p = i/m$ and $q = j/m$. Substituting $i = pm$ and $j = qm$ and taking the limit $m \to \infty$ we obtain:

$$L(p,q) = IS(i,j) - 2p^2(1-s) - q^2(1-s) + p(1-3s) + q(1-s) - \frac{(1-3s)}{2}$$

$$H(p,q) = -p^2(1-s) - \frac{q^2(1-s)}{2} + q(1-2s) + \frac{s}{2}$$

Hence, we can show that, for large $N$, the most abundant combination of strategies $(p_{opt}, q_{opt})$ is given by maximizing $L(p,q) + 2(N-1)\mu.H(p,q)$, thus we get:

$$(p_{opt}, q_{opt}) = \begin{cases} \left(\frac{1-2s}{3(1-s)}, \frac{1-2s}{3(1-s)}\right), & \text{if } 2N\mu \leq \frac{1+s}{1-2s} \\ \left(\frac{1+2N\mu(1-2s)-3s}{4(1-s)(N\mu+1)}, \frac{1}{2N\mu+1}\right), & \text{otherwise.} \end{cases} \quad (3)$$

With $s = 0$ we recover the results obtained in Rand [9]. For increasing $s$, both the optimal $p$ and $q$ decrease for small mutation rates, and for larger mutation rates only $p$ values decreases.

# 6 Extended stochastic model for the noisy AG

The acceptance probability $\alpha[p]$, mentioned in the main text (Figure 3), stipulates how likely it is that a dictator is accepted at $t + 1$ when giving $p$ at time $t$.

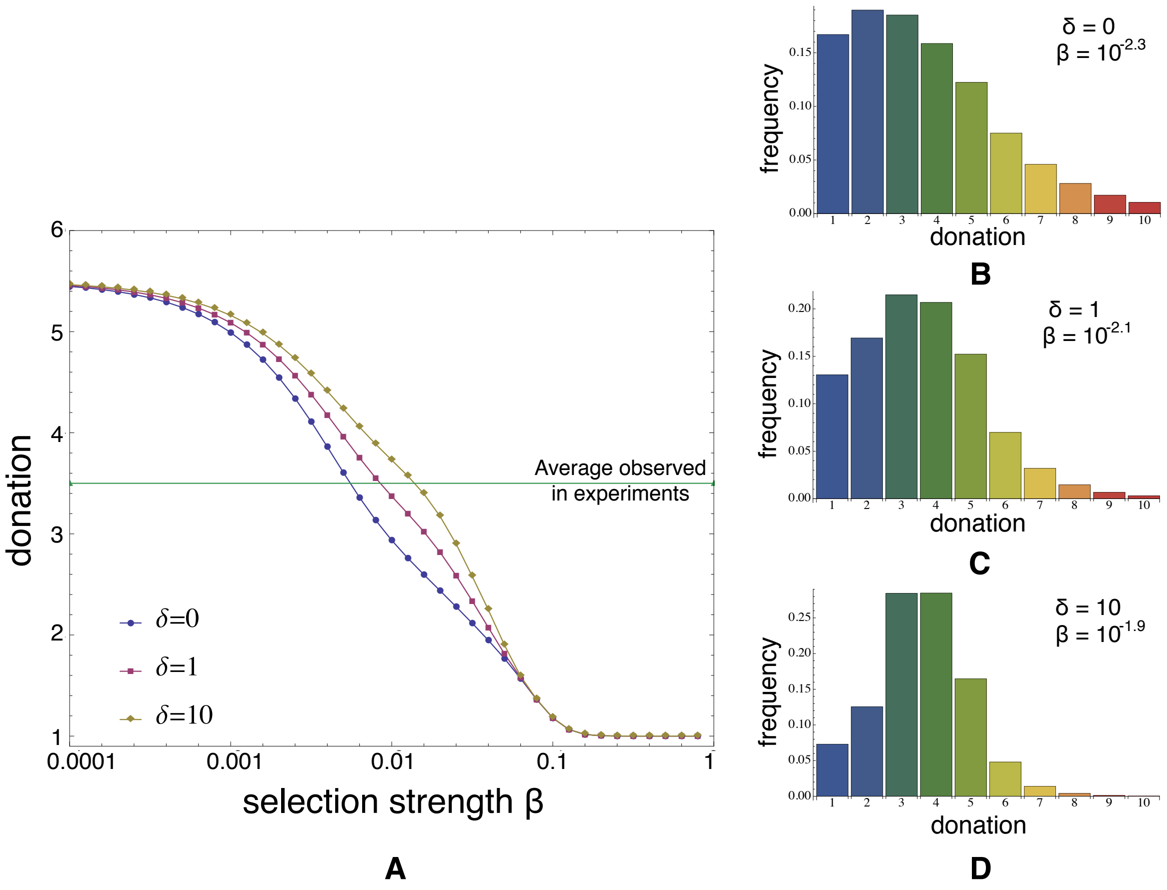


**Figure S9.** Effect of noise in the future payoff importance model. **A**. The interplay of the intensity of selection $\beta$ with the importance factor $\delta$ has the same effect in the average donations as in the AG. By increasing $\delta$ we witness more generous outcomes for a specific value of $\beta$. **B-D**. Distribution of strategies for this $\beta$ that fits best the experimental average, when future payoff importance is $\delta = \{0, 1, 10\}$. Initial endowment, $X = 10$.

The new fitness function, which takes into account also the future payoff, can also be introduced in the noisy AG model, yet in that case one also has to consider the fitness of a dictator when her donation is less than what is expected ($p < q$). In that case one obtains :

$$f_D(p) = ((X - p) \times (1 - \omega) \times (1 + \delta))/(1 + \delta) = (X - p) \times (1 - \omega) \quad (4)$$

One immediately notices that in that situation $\delta$ does not play a role. This makes perfect sense as the dictator will benefit from the payoff of the next round only if her matched receiver will not know her strategy.

The Figure S9A, which is the same as Figure 5 in the main text, shows the average donations according to the selection strength $\beta$, under low mutation rates, for varying $\delta$ parameter. We notice again (as in Figure 4 of the main paper) that the importance parameter $\delta$ promotes generosity the more it gets increased. In Figure S9B one can see the effect of $\delta$ in the distribution of dictators strategies in the noisy AG. These distributions results are intriguing as they show that for increasing $\delta$ the strategy distribution gets closer and closer to the one observed in the experiments. Hence, this result ($\delta = 10$) shows that the dictators in the experiments consider acceptance and as a consequence future gains as highly important.

Finally, in Figure S10, one may observe that our extension to the stochastic evolutionary dynamics model fits the experimental acceptance rates while maintaining the predictive capacity of the model between treatments.

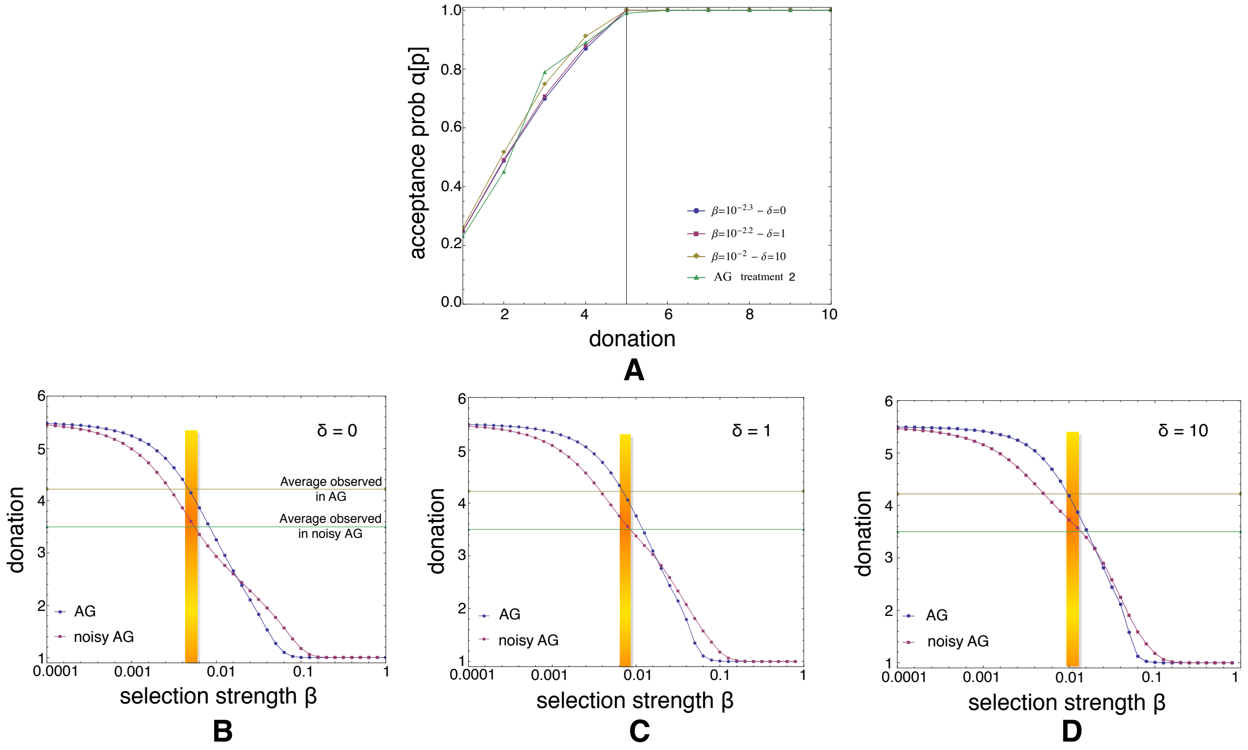


**Figure S10.** Final fitting results for the future payoff importance model. **A**. Relation between the acceptance probability $\alpha$ and the donation $p$ for those $\beta$ and $\delta$ configurations that fit best the average observed in our experiments. **B-D**. For all $3$ values of $\delta$ we tested, $0$, $1$ and $10$, we observe a range of $\beta$ that fits the average donations observed in the AG and noisy AG.

# References


[1] Fischbacher, U. z-tree: Zurich toolbox for ready-made economic experiments. *Exper. Econ.* **10**, 171–178 (2007).

[2] Camerer, C. F. *Behavioral Game Theory*. - (Princeton University Press, 2003).

[3] Nowak, M. A., Sasaki, A., Taylor, C. & Fudenberg, D. Emergence of cooperation and evolutionary stability in finite populations. *Nature* **428**, 646–650 (2004).

[4] Imhof, L. A., Fudenberg, D. & Nowak, M. A. Evolutionary cycles of cooperation and defection. *Proc. Natl. Acad. Sci. USA* **102**, 10797–10800 (2005).

[5] Nowak, M. A. *Evolutionary Dynamics: Exploring the Equations of Life* (Harvard University Press, Cambridge, MA, 2006).

[6] Traulsen, A., Hauert, C., De Silva, H., Nowak, M. A. & Sigmund, K. Exploration dynamics in evolutionary games. *Proc. Natl. Acad. Sci. USA* **106**, 709–12 (2009).

[7] Traulsen, A., Nowak, M. A. & Pacheco, J. M. Stochastic dynamics of invasion and fixation. *Phys. Rev. E* **74**, 011909 (2006).

[8] Fudenberg, D. & Imhof, L. A. Imitation processes with small mutations. *J. Econ. Theory* **131**, 251–262 (2005).

[9] Rand, D. G., Tarnita, C. E., Ohtsuki, H. & Nowak, M. A. Evolution of fairness in the one-shot anonymous ultimatum game. *Proc. Natl. Acad. Sci. USA* **110 no. 7**, 2581–2586 (2013).

[10] Ohtsuki, H. Stochastic evolutionary dynamics of bimatrix games. *J. Theor. Biol.* **264**, 136?142 (2010).
